# The Effects of Whole Body Vibration on Mobility and Balance in Parkinson Disease: a Systematic Review

**Published:** 2014-07

**Authors:** Sharareh Sharififar, Rogelio A. Coronado, Sergio Romero, Hassan Azari, Mary Thigpen

**Affiliations:** 1 Department of Physical Therapy, College of Public Health and Health Professions, University of Florida, Gainesville, FL, USA;; 2 Department of Anatomical Sciences, School of Medicine, Shiraz University of Medical Sciences, Shiraz, Iran

**Keywords:** Vibration, Balance, Parkinson disease

## Abstract

Whole body vibration (WBV) is a contemporary treatment modality that holds promise as an exercise training method in health–compromised individuals. A growing number of studies on individuals with Parkinson Disease are examining whether WBV improves balance and functional mobility. However, interpreting WBV studies is challenging since there is variability in the manner in which WBV intervention is conducted. The primary goal of this systematic review was to investigate the effect of WBV on improving mobility and balance as measured by a battery of clinical tests, in patients with Parkinson disease. Studies based on WBV parameters were characterized and a systematic search of peer-reviewed literature in five major databases was conducted. Randomized-controlled trials investigating the effects of WBV in patients with a Parkinson diagnosis and no cognitive impairment were included. A total of six publications met the inclusion criteria. Overall, studies demonstrated mixed results in favor of WBV for improving balance or mobility. The majority of studies seem to suggest a favorable benefit following WBV for mobility and balance, but not when compared to other active intervention or placebo. There was variability in the manner in which WBV intervention was applied. Variations among the six studies included: duration of intervention and rest, follow-up period, type of control groups, frequency of vibration, number of treatment sessions and sex distribution of subjects. Future research is needed to investigate the effects of different types of equipment and treatment dosage in individuals with Parkinson disease.

## Introduction

Many therapeutic interventions designed to exercise in a gym setting are currently used to improve mobility and balance in a clinical population. These exercises are characterized by different modes, intensities and demands on the individual. One exercise that may be beneficial for improving mobility and balance utilizes vibratory stimulation. The effect of vibratory stimulation on the neuromuscular system has been studied in different therapeutic and rehabilitative fields^1^^-^^5^ and has evolved into full body training known as Whole Body Vibration (WBV).^6^

WBV is targeted at individuals who have difficulty walking^7^ and who may be less inclined to participate in more vigorous training.^8^^,^^9^ WBV has been shown to improve gait and balance in patients with multiple disease conditions, such as cerebral palsy,^10^ multiple sclerosis^11^^,^^12^ and stroke.^13^ A recent systematic review and meta-analysis by Lam et al. examined the effects of WBV on outcomes related to balance, mobility and falls in older adults without known medical disease.^14^ Overall, these investigations show some evidence for improving balance and mobility outcomes, but the effects are inconclusive.

One condition where WBV may enhance mobility and balance is Parkinson disease. The effects of a vibration-type stimulus in individuals with Parkinson disease were first identified when patients displayed fewer symptoms when they were travelling on a train.^15^

The purpose of this study was to conduct a systematic review of published literature on the effect of WBV on mobility and balance outcomes in individuals with Parkinson disease. The primary aim of this investigation was to examine whether WBV studies showed a consistent positive effect on mobility and balance outcomes. It is hypothesized that WBV would have a positive effect on both mobility and balance. Further, it is intended to examine whether WBV effects on mobility and balance were greater than compared to a control intervention. Secondarily, it is sought to investigate whether studies used similar or different WBV parameters, including but not limited to variables such as characteristics of the vibration stimulus, treatment duration and overall dosage. We hypothesized that there would be variation in how WBV has been implemented in different studies. 

## Methods

*Information Sources*

A comprehensive systematic literature search was performed using the following databases: MEDLINE (PubMed), Cumulative Index to Nursing and Allied Health Literature (CINAHL), Proceedings First (limit sub headings: orthopedics), Dissertation & Theses and Sport Discus. MESH terms (PubMed) and Major Headings (CINAHL) were used when available. These databases were accessed online by May 2013.

*Systematic Search*

Key words used in the literature search included “vibration”, “whole” OR “Vibration/therapeutic use” AND “Parkinson Disease”. No limitation on language was applied. Randomized-controlled trials (RCTs) that investigated the effects of WBV on measures of mobility and balance in patients with Parkinson disease were included. RCTs that included human adult participants of any age with idiopathic Parkinson diagnosis were also considered. Study trials with healthy, older adults or adults with non-Parkinson diagnoses were excluded. 

The intervention of interest was WBV and it was operationally defined as mechanical vibration, performed with a straight body (standing or lying). Common therapeutic interventions such as localized mechanical vibration (e.g., vibration pads), ultrasound, and electrical stimulation were not recognized as the WBV. Studies where the vibration signals were not received through a completely straight body (e.g., sitting on a vibrating chair) were excluded. Acceptable comparison interventions included no treatment, sham vibration (audible sound with no mechanical vibration) and exercise. Any trials with more than two study arms were also included in the analysis without eliminating any of the arms of the study.

The outcome was not restricted to a specific outcome of interest as studies used variety of mobility and balance outcome measures, including but not limited to Unified Parkinson’s Disease Rating Scale (UPDRS), timed up and go test (TUG), functional reach test (FRT), single-leg & tandem stance tests, etc. However, studies not including a balance or mobility outcome were excluded. 

*Study Selection*

The first author screened all articles for eligibility after the search of the databases and reference lists. The initial screening step involved examining the article title and major key words. If the title and key words did not provide adequate information for inclusion, abstracts were screened. Articles that appeared to meet the inclusion criteria based on title and abstract screening were considered potentially relevant. All other articles deemed not relevant were excluded. 

*Data Extraction*

Two authors (S.S. and R.C.) extracted relevant data from each article. This data included the study design, sample characteristics (sample size, sex), intervention and control group characteristics, outcome measures and relevant results. WBV characteristics that were obtained from studies included type of vibration (stochastic or not random), frequency (Hertz), amplitude (millimeters), repetitions (number of sessions per week), bouts or cumulative dose (total number of minutes per study duration) of WBV. 

In order to assess the effects of WBV, study results were categorized based on whether “within-group” effects or an “interaction” effect was obtained. A within-group effect would be noted if the group receiving WBV showed a favorable change in outcome (i.e. pre- and post-intervention change). An interaction effect would be noted if the group receiving WBV showed a favorable change in outcome compared with the control group (i.e. greater effect from pre- to post-intervention in WBV group). Categorizing results in this manner would assist in assessing the overall findings from the included studies. 

*Study Methodological Quality*

The methodological quality of the included articles were assessed by two independent raters using the standardized and validated PEDro scale^16^ for quality of controlled clinical trials. The PEDro scale is an 11-item scale that has been previously used in systematic reviews.^17^ Agreement for quality assessment was measured with percent agreement, a Kappa statistic and 95% confidence interval (CI). Kappa values of 0.60 can show a substantial agreement between raters.^18^

## Results

*Study Selection*

**Figure 1:**
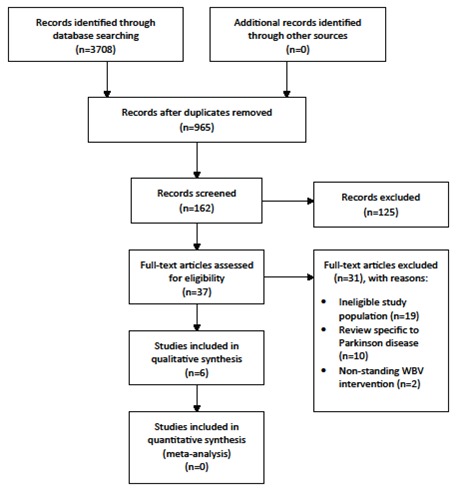
Flow diagram of search identification and selection.

Results from the systematic search are summarized in figure 1. Among 3708 identified articles, 37 were considered as having potential for inclusion, from which six were deemed as meeting inclusion criteria. Several studies were excluded for the following reasons: ineligible study population (n=19), review specific to Parkinson Disease (n=10) or non-standing WBV intervention (n=2).

*Study Characteristics*

A summary of the characteristics of included studies is presented in table 1. A total of six RCTs investigating the effects of WBV on mobility and balance were included in this systematic review. These studies included a combined number of 252 participants with Parkinson disease. Each study had a small sample size of less than 100 participants (range: 21 to 68 total participants) with the ratio of males to females being inconsistent. Males were the dominant sample in control and intervention groups in all studies. The mean age range for study participants ranged from approximately 65 to 74 years old. For all studies, individuals with Parkinson disease were included if they had Hohen–Yahr stage I-III, suggesting mild to moderate disease condition and without serious problems with balance ability.

**Table 1:** Summary characteristics of included studies (N=6)

**Study**	**Design **	**Sample**	**Groups**	**Outcome Measures**	*Within-group Effect **(Yes/No) **	*Interaction Effect **(Yes/No)**	**Conclusion**	**Quality**
Arias 2009^20^	Study Type: Double-blind, randomized, placebo controlled study	Sample: 21 patients with PD Mean age: 66.7 years Sex: 9 female	Treatment: WBV (n=11) Control: placebo (same position, no vibration) (n=10)	BBT FRT Gait analysis (i.e. velocity, cadence, etc.) TUG UPDRS	Yes	No	WBV resulted in significant improvements in mobility and balance, but not to a greater degree than placebo.	4
Chouza 2011^19^	Study Type: Single-blind, randomized, placebo controlled study	Sample: 48 patients with PD Mean age: NR Sex: NR	Treatment: WBV (random allocation to 3,6,9 Hz) (n=NR) Control: placebo (same position, no vibration) (n=NR)	FRT TUG	Yes	No	WBV at all frequencies resulted in significant improvements in mobility and balance, but not to a greater degree than placebo.	6
Ebersbach 2008^21^	Study Type: Single-blind, randomized controlled study	Sample: 27 patients with PD Mean age: 73.8 years Sex: 7 female	Treatment: WBV on oscillatory platform. (n=14) Control: conventional PT (balance board training) (n=13)	Pull test Posturography Stand –walk-sit test Tinetti Balance score UPDRS Walking velocity	Yes	No	WBV resulted in significant improvements in most mobility and balance measures, but not to a greater degree than conventional PT.	5
Hass 2006^24^	Study Type: Single-blind, randomized crossover study	Sample: 68 patients with PD Mean age: 65.0 years Sex: 15 female	Treatment: WBV Control: 15 min. rest	UPDRS (mobility only)	Yes	Yes	WBV resulted in significant improvement in mobility and to a greater degree than rest.	7
Kaut 2011^22^	Study Type: Double-blind, randomized controlled study	Sample: 36 patients with PD Mean age: 69.4 years Sex: 7 female	Treatment: WBV (n=19) Control: sham (same position with 1 HZ frequency) (n=17)	UPDRS	Yes (only for bradykinesia and postural stability)	Yes (only for bradykinesia and postural stability)	WBV resulted in significant improvements in bradykinesia and postural stability and not to a greater degree than sham.	8
Turbanski 2007^23^	Study Type: Single-blind, randomized controlled study	Sample: 52 patients with PD Mean age: 69.1 years Sex: 14 female	Treatment: stochastic WBV (n=NR) Control: rest (n=NR)	Tandem and narrow standing UPDRS	Yes	Yes (only for tandem standing)	WBV resulted in significant improvements in narrow and tandem standing, but only tandem standing effects were greater than rest.	2

*Within-group effect denotes a favorable change in outcome within the WBV group, while an interaction effect denotes a greater, favorable change in outcome compared to control. BBT: Berg Balance Test; FRT: Functional Reach Test; NR: Not Reported; PD: Parkinson disease; PT: Physical Therapy; TUG: Timed Up and Go; UPDRS: Unified Parkinson’s Disease Rating Scale; WBV: Whole Body Vibration

The parameters of WBV intervention varied across all studies, including WBV frequency, amplitude and dosage (table 2). In three of the six studies, participants received larger amplitude (e.g. between 7-14 mm) oscillations and two of these studies reported frequencies of 25 Hz.^19^^-^^21^ The majority of WBV sessions incorporated WBV bouts of 1-minute duration with 1-minute rest intervals. However, there was considerable variability in the dosage of WBV throughout the entire study. The cumulative dose of WBV ranged from as little as 5 minutes to 90 minutes. Further, participants received WBV during 1 session or up to 12 times over 5 weeks.

**Table 2:** General overview of whole body vibration parameters within each study

**Study**	**Type of WBV **	**Amplitude**	**Frequency**	**Repetitions** **(number of cycles)**	**Cycles**	*Cumulative dose
Arias 2009^20^	Non-stochastic	7-14 mm	25 Hz	12 (over 5 weeks)	5 bouts of 1 minute each	60 minutes
Chouza 2011^19^	Stochastic	13 mm	3, 6, 9 Hz	1	5 bouts of 1 minute each	5 minutes
Ebersbach 2008^21^	Stochastic	7-14mm	25 Hz	3 (over 3 days)	2 bouts of 15 minutes each	90 minutes
Hass 2006^24^	Stochastic	NR	6 Hz	1	5 bouts of 1 minute each	5 minutes
Kaut 2011^22^	Stochastic	NR	6.5 Hz	15 (over 3 days)	5 bouts of 1 minute each	75 minutes
Turbanski 2007^23^	Stochastic	3 mm	6 Hz	1	5 bouts of 1 minute each	5 minutes

*Cumulative dose is the total number of minutes of WBV for the entire study duration. Hz: Hertz; mm: millimeters; NR: Not Reported

The control interventions used both no treatment controls (i.e. rest) and groups, which received alternative exercise regimens (e.g. physical therapy) or a placebo condition that mimicked WBV. Except for the study by Ebersbach et.al, no study considered a matched time protocol between experimental and control groups. Further, the study by Ebersbach was the only study that involved an alternative form of active treatment. In this study, conventional physical therapy was used which involved training on a balance board.^21^ Placebo conditions were used as controls in three studies.^19^^,^^20^^,^^22^ The placebo, in general, involved a condition where WBV was mimicked. However, none of these studies mentioned anything related to the patient’s perception of the placebo (i.e. believability).

All six trials were of relatively short follow-up duration (less than 6 weeks) with half of the studies only examining effects after a single session.^19^^,^^23^^,^^24^ Mobility and balance outcomes varied across studies and included tests such as the Unified Parkinson’s Disease Rating Scale (UPDRS), timed up and go test (TUG), functional reach test (FRT), narrow & tandem standing, Tinetti Balance test, stand-walk-sit test, gait assessment, pull test, posturography and Berg Balance Test (BBT). No single outcome measure was used consistently across all trials. The most commonly utilized outcome was the UPDRS as it was used in 5 of the 6 trials.

*Effect of WBV on Mobility and Balance*

Overall, there was no apparent consistency in any specific mobility or balance outcome following WBV in patients with Parkinson disease. All six studies showed a within-group effect following WBV. That is, these studies showed a positive, favorable benefit following WBV in comparison to pre-test measurements. Three studies showed this effect on both mobility and balance,^19^^-^^21^ while one study showed this effect on mobility^24^ and two studies showed this effect on balance.^22^^,^^23^ An interaction effect was found in three studies.^22^^-^^24^ That is, these studies showed a greater favorable benefit following WBV as compared with the control intervention. Two of these studies used a no treatment control where participants just rested,^23^^,^^24^ while one study used a sham.^22^ The other studies showing no interaction effect compared WBV with either placebo^19^^,^^20^ or an active treatment.^21^

*Methodological Quality*

The range of scores for the trials based on the PEDro scale was 2 to 8 and suggest overall fair to moderate quality. The agreement between raters for the quality scores was moderate (percent agreement=81.82%, Kappa=0.609, 95% CI=from 0.330 to 0.888) 

## Discussion

A comprehensive and systematic review was conducted on the effects of whole body vibration on balance and mobility outcomes in individuals with Parkinson disease. Only few studies related to the effects of WBV on Parkinson disease were found that allowed scoring according to objective criteria by the PEDro scale. Of the studies that were included, there was no apparent consistency in the effect of WBV on outcomes related to mobility and balance, but a majority of these studies seem to exhibit a favorable effect following WBV. However, in general, this effect did not seem to be greater than a placebo or active treatment, but only when compared with the rest. Further, variability in the parameters of WBV was found, including differences in the types of machines used, the parameters of vibration and the duration of the interventions. Overall, the evidence for WBV use on clinical measures of mobility and balance in patients with Parkinson disease is inconclusive and highly variable.

Similar to other therapeutic interventions, WBV shows primarily a beneficial effect on outcomes when compared to receiving no treatment, but not compared with other active interventions. This effect, however, based on this systematic review, is limited to only mobility and balance outcomes and the only active intervention incorporated was physical therapy using a balance board. It is uncertain whether WBV would be more or less effective than other types of active intervention, like different forms of exercise. Furthermore, while placebo or sham control groups were also used, these results show conflicting findings. One observation was that no study assessed whether the placebo or sham condition was believable to either the practitioner or patient. Believability of placebo has been shown to be one factor that can impact the adequacy of a placebo condition.^25^ Future studies using placebo should assess this.

Regardless of the type of control utilized within the included studies, it is difficult to attribute why WBV resulted in an interaction effect in some studies and not in others. For example, in the studies, which show a preferential interaction effect of WBV (compared with control), there was no consistency in how WBV was applied. A noteworthy finding was that one of the studies that showed a larger comparative benefit (compared with a sham intervention) used WBV with a greater cumulative dosage (75 minutes) with intermittent bouts over a short period of time (3 days).^22^ However, this finding is interpreted cautiously as two other placebo-controlled studies showed no comparative benefit with dosages greater than 60 minutes.^20^^,^^21^ Thus, it seems that dosage alone does not provide a greater effect for WBV.

It is suspected that other WBV characteristics can impose differing effects on an individual’s response to the intervention. For example, some of the studies included in this review applied variable oscillation protocols.^19^^,^^23^^,^^24^^,^^26^ Brain analyzing studies have shown different activating effects of sinus waves and variable oscillations.^27^ A non-predictable stimulus activates more pre frontal areas that are known to be involved in non-routine decision-making and novel learning which are less active in patients with Parkinson disease. The random protocol used in the study by Kaut et al.^22^ found that a course of vibratory therapy was effective in improving walking ability and reducing stiffness in a group of patients with Parkinson disease. It is possible that modification in brain activation could result from random whole body vibration applied in these studies and consequently improve the individual’s postural control. Thus, a random vibration system may be more suitable for people who have bradykinesia and freezing problems while tremor is less prominently influenced.

In this review, no study assessed the additive effect of WBV on outcome. For example, it is undetermined whether combining WBV with other forms of active treatment would yield larger effects than when utilizing either treatment alone. In clinical practice, interventions are rarely applied in isolation and are commonly combined with other therapies for maximum therapeutic benefit. This has not been studied using WBV on mobility and balance outcomes. It seems as though this would be an important area of research to determine the clinical effectiveness of WBV. In order for studies to examine these effects, larger sample sizes will be needed. 

The underlying mechanisms of benefit for WBV remain elusive. Studying the underlying mechanisms is vital to understand how these interventions result in clinical improvement. Further, elucidating the parameters of WBV that most impact clinical outcomes is necessary for optimizing care. Several theories suggest that neuromuscular activation^3^^,^^4^ and metabolic mechanisms^28^^-^^31^ play a significant role in transmission of plantar surface acceleration to the weight bearing bone and muscles in both animal^30^ and humans.^28^^,^^32^^,^^33^ WBV provides a mechanical oscillation of a specific frequency and amplitude of displacement.^34^ The oscillatory vertical motion or a movement along the horizontal axis is transmitted to the whole body. While one maintains balance on the moving platform, WBV provides tactile and proprioceptive sensations to the whole body. This stimulation, in turn, bypasses Basal Ganglia circuitry which is affected in people with Parkinson disease.^33^^,^^35^^-^^38^ Consequently, these mechanisms may result in improved strength and endurance that is necessary for postural stability and gait.^34^^,^^39^^,^^40^

Future research recommendations can be made based on the results of this review. First, larger and well-designed randomized trials are required to examine the effects of different parameters of WBV (i.e. amplitude, dosage) on mobility and balance outcomes. Future trials should aim at examining longer term outcomes since the results of current studies are limited to less than six weeks. Further, as mentioned earlier, placebo-controlled trials should incorporate a measure of believability within the trial to assess the adequacy of the placebo condition. Due to the conflicting findings, it is possible that subgroup effects may need to be elucidated. For example, it is possible that some patients with Parkinson disease respond differently than others to WBV training. This effect should be examined in future studies. Additionally, future trials should examine measures of functional ability using validated items, beyond measures of gait and balance.

*Comparison with Other Systematic Reviews*

Three recent systematic reviews have examined the effect of WBV on sensory motor outcomes with cohorts including individuals with Parkinson disease.^37^^,^^38^^,^^41^ Two of these reviews discussed other common neurological diseases, while one review was specific to Parkinson disease. Overall, these reviews searched electronic databases including MEDLINE (PubMed), CINAHL, the Cochrance Library, Google Scholar, the Physiotherapy Evidence database (PEDro), the Excerpta Medica data base (EMBASE), TRIP data base, Web of Science, PsycINFO and Sport Discus spanning the time period from 1806 to 2011. While each used different key words; based on their inclusion criteria, most of the reviews used the PEDro scale to assess the quality of the published literature on the effect of WBV on physical and physiological outcomes in people with Parkinson disease. The three reviews reported fair to moderate methodological quality in the included studies. In the present review, only one additional study^22^ was considered as high quality that was not included in any of these prior reviews.

*Limitations*

There are several limitations to this review. While a systematic literature search of articles in five major databases were carried out, only databases mentioned in this article was examined and other available databases were not searched exhaustively. Therefore, current search results are limited to articles indexed in the mentioned databases. Second, only a limited number of studies were found that met author’s a priori inclusion criteria. The limited number of studies and the variability in both study design and outcomes prevented meta-analysis of the results. Thus, only qualitative data from the included studies is presented. Although a meta-analysis was not performed, it was possible to draw a general idea on quantitative results using the results coding strategy. Some of the included trials were at high risk for small-trial bias. In small trials, randomization is affected by a major threat to internal validity. For example, there may be imbalances between intervention and control groups on important prognostic factors. Thus, definitive conclusions are limited based on article sample size and potential prognostic factors. The evidence for a clinical benefit following WBV should be cautiously considered. 

## Conclusion

WBV has demonstrated limited, but beneficial effects on balance stability and mobility in individuals with Parkinson disease. The influence of different types of equipment and parameters on clinical outcome is undetermined but may play a role in the conflicting results. Consistency in WBV parameters that show beneficial effects is encouraged. Future studies on WBV, especially on how parameters relate to clinical outcome, could pave the way for larger and more clinically meaningful effects following WBV. 
